# Areca use among adolescents in Yap and Pohnpei, the Federated States of Micronesia

**DOI:** 10.1186/1477-7517-10-26

**Published:** 2013-10-17

**Authors:** Peter Milgrom, Ohnmar K Tut, John Gilmatam, Marcelle Gallen, Donald L Chi

**Affiliations:** 1 Northwest Center to Reduce Oral Health Disparities, University of Washington, Box 357475, Seattle, WA, USA; 2 Wa'ab Community Health Center, Colonia, Yap State, Federated States of Micronesia; 3 Pohnpei State Hospital, Kolonia, Pohnpei, Federated States of Micronesia

**Keywords:** Areca, Betel, Adolescent, Oral mucosa, Micronesia, Substance-related disorders

## Abstract

**Objective:**

Areca (Betel Nut) is the fourth most commonly used psychoactive drug throughout the world and is legal in U.S. It is carcinogenic. Within a health program in the Federated States of Micronesia we surveyed use among adolescents.

**Methods:**

One hundred 7^th^ and 8th graders in Yap and Pohnpei were surveyed and clinical oral examinations conducted. The questionnaire included items on Areca: age first used Areca, whether peers or family members used Areca, frequency of use, whether tobacco was used, and source of Areca. Questions also assessed anxiety and depression. Two scales assessed getting along with other kids and adaptation to school.

**Results:**

61.5-71.4% of adolescents had ever used Areca and 54.7-68.6% used it at least once in the last month.

**Conclusion:**

Most adolescents surveyed in Yap and Pohnpei used Areca, which may place these youth at increased risk for cancer and health disparities.

## Introduction

*Areca catechu* (Areca nut, Betel nut) is a CNS stimulant and carcinogen [1-3]. Depending on the culture, the green or dry palm nuts are crushed and chewed alone, or cut up with slaked lime and tobacco and wrapped in the leaf of the *Piper Betel vine* that is held like smokeless tobacco in the cheek. Areca nut is legal in the United States. The addition of tobacco enhances the CNS effects and lime increases absorption. Areca nut is the fourth most commonly used psychoactive drug in the world [4,5]. It has long been of social, cultural, and economic importance [6]. Areca nut use is thought to be protective against tooth decay [7].

Large proportions of adolescents in the United States Affiliated Pacific Islands (USAPI) use Areca nut [7-11]. In this setting the nut is typically used while green. The tobacco is obtained from cigarettes and may be dipped in vodka by some children before use. The lime is from baked coral. The combination is wrapped in the fresh Betel leaf. It was recently added to the United States National Survey of Drug Use and Health because of concerns that use is growing on the mainland.

Areca nut use has been posited to have social consequences, including low academic performance and poor quality of life [12,13]. A report from the United Kingdom suggested that children use Areca nut to suppress hunger [14]. Factors that might explain Areca nut use are largely unstudied [6,15]. Thus, the states of Yap and Pohnpei in the Federated States of Micronesia (FSM) conducted a preliminary public health survey of Areca nut use among adolescents.

## Methods

### Setting

The study was conducted in Yap (population 11,376) and Pohnpei (population 35,981) States, FSM. FSM is an independent country in free association with the United States. The States differ in that Yap has a long cultural tradition of adults using Areca nut and it is grown there. Use and cultivation of Areca in Pohnpei began in the last two decades. Areca nut is exported from both States [6].

### Study participants

After parent consent and child assent, 24 Seventh and 41 Eighth grade students in Yap from Colonia Middle School, the largest middle school on the island, were surveyed. This represented 84% of the children in these grades based on school records and included all the children in attendance that day. In Pohnpei, 15 Seventh and 20 Eighth grade students from Nett Middle School completed the survey. The Pohnpei sample represented 17% of the children in those grades based on school records and was a convenience sample because not all the children were available. The surveys were administered in November 2012 and were confidential. The children speak and write English. The Institutional Review Board of the University of Washington approved analysis of de-identified surveys.

### Questionnaire

Seven items regarding use were modeled after earlier work [16]. These items captured whether the individual had ever used Areca nut, age he or she first tried Areca nut, whether peers or family members used Areca nut, times per month the individual used Areca nut, whether tobacco was used with the Areca nut, and source of the Areca nut (family, friends or a shop). Four questions assessed anxiety and depression [17]; six questions assessed getting along with other kids and adaptation to school (paying attention, forgetting things, keeping up with school work). The items for each concept were summed to give an overall score where a higher score indicates greater problems. These questions were in Likert-like format with 5 choices ranging from "never a problem" (score 0), “almost never a problem”, “sometimes a problem”, “often a problem” to "almost always a problem” (score 4). The scales measuring getting along with other children and adaptation to school were combined for analysis. The possible range for the anxiety and depression scale was 0 to 16. The possible range for the combined getting along/adaptation to school scale was 0 to 24. Cronbach alphas were .68 for the anxiety and depression scale and .64 for the combined scale.

### Clinical screening

Clinical oral examinations were conducted by paraprofessional trainees after training by experienced dentists and assessed inflammatory (red or white) changes in the cheeks, tongue or gingiva; stains on the teeth, dental caries; soft tissue abscesses caused by dental caries. Standard indices were not used in this setting.

### Data analysis

The data were analyzed using SPSS Statistics version 19. The results from each community were analyzed separately. Chi-square was used to examine the relationship of peer and family use on use by the study youth. Similarly chi-square was used to explore the hypothesis that Areca nut use was protective against dental caries. T-tests were used to explore the hypothesis that individuals who used Areca nut were more likely to report anxiety and depression, poorer relationships with peers, or problems with school.

## Results and discussion

### Areca nut use

Yap: Forty of 65 adolescents surveyed (61.5%) had ever used Areca nut; 54.7% used it at least once in the last month (Table 1). There was no significant difference in any use or frequency of use among users between sexes (Chi-square = 1.31, df 1, p > .05). The median age a child first used Areca nut was 11 years and one-in-four had used it by age 9 years. Ten of 40 (25%) used tobacco with the Areca nut most of the time. Eighteen of 40 (45%) said they did not use tobacco with the Areca nut. Those who used tobacco also used lime. Peer influences were strong: 54.7% of those who used Areca nut during the last month reported that their best friends at school or in the village used Areca nut, but the relationships were not significant (Chi-squares = .014 and 2.7, respectively; df 1, p > .05). Similarly, over 50% of students reported Areca nut use among their mothers and fathers, but there was no association between parental and adolescent use.

**Table 1:** Areca nut use and frequency among adolescents in Yap (N = 65) and Pohnpei (N = 35), federated states of micronesia

**Question**	**Yap n (%)**	**Pohnpei, n (%)**
Have you ever used Betel		
Nut (Areca)?		
Yes	40 (61.5)	25 (71.4)
How often did you use Betel		
Nut (Areca) in the last		
month?		
*Not at all*	29 (45.3)	11 (31.4)
*Once a month*	2 (3.1)	3 (8.6)
*Once a week*	16 (25.0)	2 (5.7)
*2-3 times per week*	2 (3.1)	7 (20.0)
*4-5 times per week*	4 (6.3)	3 (8.6)
*Every day*	10 (15.6)	5 (14.3)

Pohnpei: 25 of 35 adolescents (71.4%) had ever used Areca nut; 69.6% used it at least once in the last month (Table 1). There was no significant difference in any use or frequency of Areca nut use among users between the sexes. The typical child first tried Areca nut at age 11 and one-in-four tried it by age nine. Ten of 25 (40%) used tobacco with the Areca nut most of the time. Only four of 25 current users (16%) said they did not use tobacco with the Areca nut. Those who used tobacco also used lime. Peer influences were strong: 48.6% and 62.9% of those who used Areca nut reported that their best friends at school or in the village used Areca nut, respectively, but the relationships were not significant (Chi-squares = .013 and 2.2, respectively; df 1, p > .05). There was no association between parental and adolescent use.

In both states, almost all Areca nut users reported obtaining the Areca nut from members of the family (24/69) or from school or village friends (39/69). It was rarely purchased (5/69).

### Clinical findings

Areca nut use among all youth was associated with stains on the teeth but mucosal changes were uncommon. Twenty-one of 61 adolescents (34.4%) in Yap had serious decay or an abscess. Twenty-four of 34 adolescents (70.6%) in Phonpei had serious decay or an abscess. Areca nut users in Yap were less likely to have serious decay but the difference was not significant (13.1% versus 21.3%, p = .069). Areca nut users in Pohnpei were more likely to have serious decay (50% versus 20.6%) but the difference was not significant (Chi-square = .379, df 1, p = .53).

### Mental health, relations with peers, and school

The mean (SD) anxiety and depression scores were 6.8 (3.8) and 6.7 (3.6) in Yap and Pohnpei, respectively, where the ranges were from 0 to 16 and 0 to 14, respectively, and the maximum possible score was 16. The combined mean (SD) score for getting along with peers/school adaptation in Yap was 7.62 (4.94) where the range was from 0 to 21 (maximum possible score 24). The combined mean (SD) score for getting along with peers/school adaptation in Pohnpei was 7.14 (2.48) where the range was from 2 to 12 (maximum possible score 24). Higher scores indicate greater problems. There were no differences in the scores in either State when t-tests were used to compare those who have used Areca nut in the last month versus others.

## Discussion

Our data indicate about half (32/65 in Yap and 17/35 in Pohnpei) used Areca nut once per week or more in the last month. Little is known about whether Areca nut use, with and without tobacco, leads to the development of tolerance among youth or the need to use it more frequently to obtain the same CNS effects. Limited research on adults suggests that chronic users of Areca nut alone develop dependence [18]. Nearly twice as many adolescents in Pohnpei as Pohnpei who use Areca nut at all also used tobacco with it most of the time (40% versus 23.8%), and clearly tobacco users develop dependency [19]. Although most adolescents obtained their Areca nut from family or friends, there was no association between peer or parental use and adolescent reports of use. This is in contrast to findings on cigarette smoking where peer influences are very strong [20].

Clinically examiners saw clinical changes (stains and apparent burns) on the lower lips of users who also used slaked lime and tobacco but few of the participants had inflammatory changes. We found no consistent protective effect of chewing on tooth decay.

Adolescent perceptions of risk of Areca nut use have not been measured. We know from study of other substance use that youths tend to underestimate risk. The literature from Taiwan [16] suggested that mental health and school problems might be higher in the population of adolescent Areca nut users. However, we found no association between depression and anxiety, peer relationships, or adaptation to school among these Micronesian adolescents. Nevertheless, the population studied is a relatively small convenience sample and the youth are young. These problems might reveal themselves in larger samples of older youths or adults.

## Conclusions

These findings indicate need for research to identify the determinants of Areca nut use in Pacific Islander communities. Such research should adopt multilevel conceptual frameworks focusing on individual-, family-, peer-, and community-level factors that influence the initiation and maintenance of Areca nut use. The knowledge can be used to develop community-based interventions and policies aimed at reducing Areca nut use among youth.

## Competing interests

The authors declare that they have no competing interests.

## Authors’ contributions

PM conceptualized the study, analyzed the data, and wrote the first and final drafts of the manuscript. OKT trained the clinical examiners and contributed to the final manuscript. JG arranged for the cooperation of the schools on Yap and contributed to the final manuscript. MG arranged for the cooperation of the schools on Pohnpei and contributed to the final manuscript. DLC helped conceptualize the research and analysis and participated in the interim and final draft of the manuscript. All authors read and approved the final manuscript.
